# Beyond DNA: the Role of Epigenetics in the Premalignant Progression of Breast Cancer

**DOI:** 10.1007/s10911-018-9414-2

**Published:** 2018-10-10

**Authors:** Rebecca S. DeVaux, Jason I. Herschkowitz

**Affiliations:** Department of Biomedical Sciences, Cancer Research Center, University at Albany, State University of New York, Rensselaer, NY USA

**Keywords:** Breast cancer progression, DCIS, IDC, Epigenetics, DNA methylation, Histone modification, Noncoding RNA, Enhancer

## Abstract

Ductal Carcinoma in Situ (DCIS) is an early breast cancer lesion that is considered a nonobligate precursor to development of invasive ductal carcinoma (IDC). Although only a small subset of DCIS lesions are predicted to progress into a breast cancer, distinguishing innocuous from minacious DCIS lesions remains a clinical challenge. Thus, patients diagnosed with DCIS will undergo surgery with the potential for radiation and hormone therapy. This has led to a current state of overdiagnosis and overtreatment. Interrogating the transcriptome alone has yet to define clear functional determinants of progression from DCIS to IDC. Epigenetic changes, critical for imprinting and tissue specific development, in the incorrect context can lead to global signaling rewiring driving pathological phenotypes. Epigenetic signaling pathways, and the molecular players that interpret and sustain their signals, are critical to understanding the underlying pathology of breast cancer progression. The types of epigenetic changes, as well as the molecular players, are expanding. In addition to DNA methylation, histone modifications, and chromatin remodeling, we must also consider enhancers as well as the growing field of noncoding RNAs. Herein we will review the epigenetic interactions that have been uncovered in early stage lesions that impact breast cancer progression, and how these players may be utilized as biomarkers to mitigate overdiagnosis and overtreatment.

## Background

Increased emphasis on breast cancer screening and advancements in imaging technology over the past 30 years have led to a dramatic increase in diagnosis of early stage precursor lesions [1, 2]. The current model of breast cancer progression suggests several stages of nonobligate precursor lesions with a linear progression from atypical ductal hyperplasia (ADH) to ductal carcinoma in situ (DCIS), characterized by growth of abnormal cells contained within the mammary duct, to invasive ductal carcinoma (IDC) where the cells gain invasive capacity and expand beyond the duct [3, 4]. Today 1 in 4 of all breast cancers diagnosed are diagnosed as DCIS, however only a small subset are likely to progress into invasive disease [5–7]. Currently, the molecular mechanisms underlying progression from DCIS to IDC are poorly understood and distinguishing those DCIS likely to progress from innocuous lesions remains a critical clinical challenge. Thus, patients diagnosed with DCIS will nearly all undergo surgery, with the potential of radiation and hormone therapy as well. This has created a current state of overdiagnosis and overtreatment. In fact the U.S. Preventative Services task force found that “…for every woman who avoids a death from breast cancer through screening, 2 to 3 women will be treated unnecessarily [6].”

While transcriptomic and proteomic studies have identified some genes that clearly change between DCIS and IDC [8–11], the majority of transcriptome changes are found in the transition from normal epithelia to ADH or DCIS. The profiles of the transition from DCIS to IDC are found to be highly similar to nearly indistinguishable, depending on the study, suggesting changes that drive invasive potential are already present in DCIS [12–17]. Studies of early stage lesions are frequently limited by small sample size and identification of candidate genes frequently varies between studies. Thus, to date no clear functional determinant or signature to distinguish DCIS likely to progress has emerged. To complement transcriptomic studies, the impact of epigenetic changes are under investigation for their role in early disease progression.

Originally defined in 1942 as a controversial catchall for unexplained phenotypes induced by stress, epigenetic changes are defined as stable changes to a phenotype without changes to the genotype. Epigenetic mechanisms work to engage gene expression programs, pass down inheritable traits, and define tissue specific expression programs all without altering the underlying DNA sequence [18–20]. Eighty years later, control of gene expression due to DNA methylation and histone modification have become two of the most well studied epigenetic modifications. However, technological advances continue to reveal new molecular players including enhancers and noncoding RNAs. Herein we will review how epigenetic modifications may be critical to driving early stage breast lesions to invasive carcinomas.

### DNA Methylation

DNA methylation, a covalent and reversible DNA modification, was recognized to play a functional role in gene expression in the 1980s [18, 21]. The formation of 5-methyl-cytosine (5-me-C or m5C), through the addition of a methyl (CH3) group to the 5th carbon of the pyrimidine ring of a cytosine residue, is found to primarily occur at CpG islands. This reversible mark can be transferred by several enzymes including the polycomb repressive complex and DNA methyltransferases (DNMT1, DNMT3A, DNMT3B). CpG islands, ~1000 base pair regions of DNA with a high G-C content, are generally associated with gene promoters and their methylation is tightly linked to gene transcription [22, 23]. Dense methylation of CpG islands functionally silences the associated promoter and represses transcription by blocking transcription factor binding and recruiting chromatin remodeling complexes such as histone deacetylases (HDACs) or polycomb repressive complexes (PRC). Recruitment of HDACs remodels the local chromatin encouraging closed heterochromatin formation. Similarly, the recruitment of the PRC results in histone methylation and heterochromatin formation. The global loss of m5C in a variety of malignant tumor types relative to either normal tissue or benign lesions, was first observed in 1983 and was the first epigenetic change to be described in human cancer [24, 25]. While hypomethylation is a general characteristic of many cancers, including breast [26], a number of individual genes become hypermethylated. These genes are generally tumor suppressors or play critical roles in regulation of a number of hallmarks of cancer including apoptosis and cell-cycle regulation. A number of studies have examined DNA methylation in breast cancer progression.

Verschuur-Maes et al. examined the methylation status of 50 candidate tumor suppressor genes in normal epithelia, columnar cell lesions (CCL), grade I DCIS, CCL paired with DCIS and CCL paired with IDC. When considering all 50 candidates, a significant increase in cumulative methylation was found when comparing normal tissue to either CCL, DCIS or IDC, however there was not a significant global change between DCIS and IDC. However, two tumor suppressors, CACNA1A and MGMT, demonstrated an increase in methylation between DCIS and IDC. Many of the tumor suppressors studied had significant methylation even in CCL underscoring methylation as an early event [27]. Park et al. identified 15 loci differentially methylated between matched IDC and normal tissue. These loci were then tested in an additional patient cohort comprised of normal, flat epithelial atypia (FEA), ADH, DCIS and IDC. This study revealed a stepwise increase in the total number of methylated candidate genes from normal to atypia to DCIS. Several demonstrated increased methylation between normal and ADH (APC, DLEC1, HOXA1, and RASSF1A), however only two were differentially methylated in the transition from DCIS to IDC, HOXA10 and SFRP1 [28].

van Hoesel et al. employed laser capture microdissection to normal epithelia, ductal hyperplasia, ADH, DCIS and IDC samples and identified increased methylation from normal tissue to ADH for both (RARβ, RASSF1A) and from ADH to DCIS for RASSF1A. Both candidates demonstrated a significant linear trend with progression, however did not show a significant change in methylation between DCIS and IDC [29]. Also using lasercapture microdissection, Lehhman et al. found methylation of RASSF1A, and cyclin D2 to increase between normal and DCIS but were similar between intraductal and invasive samples [30]. Fackler et al. also identified increased methylation in cyclin D2, RASSF1A, RARβ, but also in TWIST and HIN-1 when comparing reduction mammoplasties to DCIS [31]. Hoque et al. evaluated methylation status in nine putative tumor supressor genes in synchronous preinvasive (either ADH or DCIS) and IDC lesions as well as pure IDC and normal tissue samples. This study identified APC, CDH1 and CTNNB1 with elevated methylation in IDC relative to normal with only CDH1 showing increased methylation in IDC compared to preinvasive lesions [32]. From these studies and more [33–36] a model is emerging wherein epigenetic DNA methylation changes are an early event in breast cancer occuring prior to development of invasive growth.

Although a few candidate genes have been identified by multiple studies (RASSF1A, RARβ, CCND2), identification of reproducible, clinically tractable DNA methylation biomarkers from previous studies is limited by differences in methodology (sample collection, statistical cutoffs), small sample size lacking statistical power, and interrogation of limited target gene panels. Improved sequencing technologies have now allowed the interrogation of the methylome. In one of the first studies to look at the global methylation profile, Tommasi et al. interrogated six frozen DCIS and adjacent normal samples and found 108 loci differentially methylated with a significant enrichment of homeobox genes. Further, 53% of identified loci were also Polycomb repressive complex targets [37]. Combining the power of array technology with a longitudinal study, Johnson et al. [38] used the Illumina HumanMethylation450 microarray to evaluate 40 estrogen receptor positive DCIS cases and adjacent normal where available (*n* = 15). 33% of initial DCIS cases went on to develop invasive disease which were then assessed versus age matched non invasive disease. This longitudinal study revealed 641 progression-associated differentially methylated CpGs. Of these, 276 demonstrated increases in methylation from normal to DCIS to progressed IDC. From the 641 loci, relating to 397 genes, 72 genes demonstrated differential methylation in an independent DCIS-IDC cohort. From this group there was a strong enrichment of homeobox genes, as well as polycomb group gene targets. Interestingly, they also identified HOTAIR*,* a long noncoding RNA that stimulates invasion and metastasis in breast cancer, as being differentially methylated and demonstrating a positive expression correlation with methylation. HOTAIR (HOX transcript antisense RNA) epigenetically silences genes through redirecting the polycomb repressive complex 2 (PRC2) to many loci including the HOXD cluster thus suggesting one potential mechanism by which both homeobox genes and PRC2 targets may be effected. Two independent studies suggest homeobox genes and polycomb target genes as critically altered during early breast cancer progression. This observation is further strengthened by a recent study where Cai et al. used the MMTV-PyMT mouse model to study DNA methylation during progression. For this study, samples were collected at specific time points corresponding to tumor progression (hyperplasia at 6 weeks, adenoma / mammary intraepithelial neoplasia at 8 weeks, early carcinoma at 10 weeks and late carcinoma with metastasis by 12 weeks). This study revealed that of 374 genes demonstrating increased methylated promoters unique to late stage samples, there was a strong enrichment for PRC2 targets. Furthermore, the authors found significantly reduced expression of PRC2 target genes at all stages of progression, suggesting PRC2 alterations as critical to early progression [39].

Although three independent studies and models have identified polycomb target genes as a group to be altered in progression, this observation requires further validation as it was not observed in all methylome studies. Fleischer et al. interrogated 285 archived tissue samples, including normal, DCIS, DCIS-IDC mixed and IDC using the Illumina Infinium HumanMethylation450 microarray. The authors also correlated methylation with gene expression. While nearly 17,000 CpGs (1011 genes) were differentially methylated between normal and DCIS, only 2000 (154 genes) were altered between DCIS and IDC. These results were validated through over 500 TCGA breast cancer samples as well as an additional set of DCIS / adjacent normal samples. Interestingly, 4 genes demonstrated increased methylation from normal to DCIS and DCIS to IDC (CPA1, CUL7, LRRTM2, and POU2AF1). The authors were also able to study methylation in relation to patient survival This study resulted in development of a prognostic signature of 18 CpG loci,correlating to 26 genes, that can predict survival of patients with IDC, DCIS and mixed DCIS-invasive lesions [34]. Interestingly, these genes were not significantly enriched for canonical signaling pathways and this signature does not include the 4 genes that increased with progression [34].

Several studies suggest that investigating the surrounding normal adjacent tissue may be critical to understanding how DCIS ultimately gain the capacity to invade. Teschendorff et al. [40] recently compared the DNA methylome of 569 breast tissue samples including patient matched DCIS – adjacent normal, as well as 50 samples from cancer free women. This study revealed dramatic changes between normal, cancer free tissue, and normal adjacent tissue demonstrating wide spread DNA methylation field effects in agreement with previous studies [41]. Interestingly, the methylation patterns in the adjacent normal were found strongly enriched at genetic regulator elements, EZH2 and SUZ12, members of the PRC2 complex, as well as CTCF and RAD21, proteins critical to chromatin looping [40]. Together these data suggest that chromatin remodeling in adjacent normal tissue is critical to progression.

DNA methylation clearly plays a role in early breast cancer progression, with the majority of changes occuring between normal and even the earliest atypical lesions. Now advances in microarray technology, and an increased emphasis on understanding progression, changes between DCIS and IDC are beginning to emerge. Globally, homeobox genes and polycomb complex genes may be critical targets to understanding progression, however this needs to be further validated in additional patient cohorts. With a focus on understanding DNA methylation in progression, and the possibilty of longitudinal patient studies, the prospect of building a diagnostic signature to predict invasive potential that incorporates DNA methylation seems within reach.

### Histone Modification

Histone modification is critical to gene regulation both through working hand in hand with DNA methylation as described above, and independently. The chromatin compaction model illustrates how DNA must be organized in nucleoprotein complexes to fit within the nucleus. Negatively charged DNA associates with an eight histone protein complex called a nucleosome. Each nucleosome is composed of two sets of histones H2A, H2B, H3, H4 and a linked histone protein H1. DNA connects nucleosomes, like beads on a string, which must then become further compacted to form tight chromatin fibers. Tightly wound DNA of heterochromatin is unavailable to transcriptional machinery thus compaction regulates gene expression. Post-translational modification of histones that alter the association of DNA with nucleosomes can alter gene expression without changing the underlying DNA [42].

Histone proteins have tails extending out of the nucleosomes that are available for post-translational modification. Histone tails are the subjects of several modifications including mono- di- or tri-methylation, acetylation, phosphorylation and ubiquitination. Each modification has a unique effect on its surroundings. Modifications can create docking sites for recruiting proteins to the nucleosome, or remodel the chromatin to either relax or tighten the DNA changing the chromatins accessibility. Multiple methylation marks at different locations are associated with different outcomes. For example, mono-methylation of H3K9, H3K27 or H3K79 is generally associated with gene activation whereas di-methylation is generally repressive on H3K9 and H3K27, but activating on H3K79. Further, several modifications can occur on the same tail and on several histones within the same nucleosome, each having an independent effect on how the local DNA associates with the histones. This complex system is governed by a series of “writers” to deposit the modifications, “readers” to interpret and “erasers” as histone modifications are reversible allowing for genome plasticity. Similar to the genetic code, we are now learning how the histone code functions in the cell to regulate gene expression and signaling networks [43, 44].

A number of studies of histone modification are focused on specific histone readers, writers or erasers to determine their impact on breast cancer. The Polycomb Repressive Complex 2 (PRC2) is a histone modification writer that plays an important role in development, stem-cell plasticity, differentiation and maintaining cell identity. PRC2 is recruited to chromatin where the catalytic subunit, enhancer of zeste homolog 2 (EZH2), a histone lysine N-methyltransferse, tri-methylates histone H3 at lysine 27, a repressive mark. EZH2 can then also serve as a scaffold for the additional recruitment of DNA methyltransferases functionally reinforcing a closed chromatin state and transcriptional repression [45, 46]. Inappropriate chromatin remodeling can result in dysregulated signaling that can cause a cell, in effect, to “forget” appropriate cell identity.

Interrogating EZH2 as the functional PRC2 subunit, tissue microarray [47] and immunohistochemistry analysis [48] of normal, atypia, DCIS, invasive and metastatic samples found EZH2 expression to increase with each stage of progression. Consistent with observations with DNA methylation, EZH2 transcripts and protein were found elevated in DCIS lesions while largely absent from normal tissue, again suggesting this is an early event. Kleer et al. interrogated the relationship between EZH2 expression and clinical outcomes and found elevated EZH2 to be associated with a more aggressive breast cancer and shorter metastasis, disease free, and overall survival [47]. Furthermore, exogenous expression of EZH2 in normal immortalized H16N2 cells increased anchorage independent growth and invasion [47]. Together this suggests that EZH2 expression and chromatin remodeling drives breast cancer progression.

Lysine-specific demethylase 1 (LSD1) is a histone methylation eraser that specifically works on H3K4 and H3K9 [49–51]. LSD1 is found highly expressed in several tumor types including prostate [52], lung [53], bladder [54] and breast [50]. Serce et al. extended this analysis to examine early breast cancer progression. When comparing low, intermediate and high grade DCIS to invasive breast cancer, LSD1 expression increases with grade, again an indication of an early epigenetic event in breast cancer progression [51]. LSD1 expression was found highest in those tumors that were estrogen receptor negative (ER-) and progesterone receptor negative (PR-) [50].

Another family of histone modifying proteins are the acetylation readers, writers and erasers. Acetylation by histone acetyltransferases (HATs) occurs on the ε-amino groups of lysine residues, effectively neutralizing the positive charge on lysine. Strong acetylation induces an open chromatin confirmation allowing transcription factor binding. Acetylated histones can also serve as a mark for recruiting bromodomain proteins (proteins that bind acetylated lysine residues, “readers”) which in turn recruit the mediator complex required for transcription activation [55–57]. Histone deacetylases (HDACs) oppose this process, remove the acetylation, and generally support a closed chromatin confirmation. To date, there are 18 HDAC enzymes divided into 4 classes based on their mechanism of action (zinc or NAD+ dependent) and location [58]. Following the discovery of HDACs in 1996 [59], the HDAC inhibitors that followed were found to attenuate cell proliferation, induce differentiation of both normal and breast cancer cell lines, and regulate expression of cell cycle genes [60–62].

Given the dramatic cell biological phenotypes of the HDAC inhibitors, many groups began investigating the functional role and expression of HDACs. Suzuki et al. investigated HDAC1, HDAC2, HDAC6, acetylated H4, and ac-H4K12 in pure normal (from reduction mammoplasties), adjacent normal, DCIS and IDC patient samples. Acetylation was largely consistent between pure normal and adjacent normal, however there was global hypoacetylation of histone H4 as well as a decreased expression of HDACs 1, 2, and 6 with progression. The most dramatic loss of ac-H4 occurred between normal and DCIS, again suggesting an early event in disease progression [63]. Recently, Lapierre et al. examined HDAC9 expression in the MCF10a breast cancer progression series which models the transitions between normal, ADH, DCIS and IDC [64–68]. HDAC9 expression was found to increase 10–12 fold between normal and DCIS model cell lines with no change between DCIS and IDC, again indicating an early event in progression [69]. While these studies suggest that HDAC expression and activity may be altered in early lesions, how HDACs may support progression remains unclear. Future studies that focus on HDACs in early lesions should also identify changes in where modifications are occurring as opposed to global changes, to inform what genes are impacted by chromatin remodeling.

To date there are very few studies focused on histone modifications in early breast cancer lesions. The majority of histone modification studies have been performed on invasive disease where many groups have found specific modifications, and associated proteins, differ based on breast cancer subtype and can correlate with tumor phenotypes and clinical outcomes. Elsheikh et al. used 880 well-characterized breast carcinomas to perform tissue microarrays for a number of epigenetic marks. Acetylation of H4K16ac was found absent from the majority of samples suggesting that loss may be an early event in breast cancer [70]. The authors were able to couple modification status with clinical outcomes and found that low detection of several modifications (H3K18ac, H3K9ac, H4K12ac, H4K16ac, H4R3me2, H3K4me2, H4K20me3) was associated with adverse overall and disease free survival [70]. Expression HDAC1 is positively correlated with positive ER/PR status, and luminal A subtype [71–74]. Seo et al. found no correlation between HDAC1 and overall survival using univariate analysis, however high HDAC1 expression was positively correlated with overall survival in ER+ patients [73]. Concordantly, while Zhang et al. also found patients with high HDAC1 expression had better patient outcomes, expression was not an independent prognostic indicator for overall or disease free survival [74]. HDAC2 expression is significantly correlated with HER2 overexpression and a negative hormone receptor status [72], and HDAC6 expression is associated with luminal B subtype [73]. Although few studies are focused on early progression, these data demonstrate that histone modification and chromatin remodeling play a clear role in breast cancer, likely as an early event in disease progression.

### Enhancers

Advances in high throughput array and sequencing technology (ChIP-seq, RNA-seq, DNAse1-seq, ChIA-Pet, Hi-C chromatin capture…) have made interrogating the transcriptome and genomic architecture more accessible, resulting in an explosion of genome wide studies. The advanced genomic approaches have both increased our understanding of the complex systems regulating transcription, and added new players to the transcriptional models. Although enhancers were originally defined in 1987 [75], our understanding of how they function and regulate gene expression is continuing to evolve.

Classic enhancers are stretches of DNA to which transcription factors bind to induce expression of target genes. Enhancers can promote local gene transcription or undergo chromatin looping to promote distal gene transcription, potentially up to several thousand base pairs away. These elements are identified by regions of DNase1 hypersensitivity, demonstrating open chromatin confirmation, associated protein binding (CTCF, Med1, CBP300), and histone modification (H3K4me1, H3K27ac). Multiple enhancers can be clustered together creating a region of extremely high occupancy of transcriptional machinery such as the Mediator complex, and an abundance of transcription factor binding sites. These clusters of enhancers (or super-enhancers) play essential roles in driving expression programs that define cell identity and are frequently tissue type specific. Importantly, their normal function can become co-opted during tumorigenesis [76, 77].

Several groups have recently reported the acquisition of super-enhancers at oncogenes during tumorigenesis [76–80]. Hnisz et al. sought to understand if acquired super-enhancers amounted to background noise or if they functionally contributed to oncogenic signaling networks. To ask this question the authors examined normal mammary epithelia compared to the ER+ MCF7 cell line through ChIP-seq for H3K27ac and found an active super-enhancer at the estrogen receptor (ESR1) locus only in the MCF7 cells [76]. Similarly, Rhie et al. identified global similarities and differences between poised and active enhancers in normal immortalized human mammary epithelial cells (HMEC) and triple negative breast cancer MDA-MD-231 cells [81]. The authors found the cell type specific enhancers in the HMECs to be associated with epidermis development, cell adhesion, wound healing, chemotaxis and proteolysis, whereas the enhancers in the TNBC cells were related to driving *chromatin modification*, DNA replication, cell division, cell cycle, and mitosis. The authors further examined their identified gene signatures in TCGA and found 60 of the MDA-MB-231 specific genes were in the top 10% of genes overexpressed in tumor versus normal, while 53 of the HMEC specific genes were in the top 10% of the underexpressed genes [81]. Together these data suggest a penetrant and dramatic shift in enhancer rewiring as a critical step in breast cancer progression.

The rewiring of super-enhancers, has recently become a new target of epigenetic therapeutic approach. Active enhancers, marked by strong chromatin acetylation, recruit bromodomain and extraterminal (BET) family proteins to facilitate gene transcription. The BET inhibitor JQ1, targeted against the bromodomain protein BRD4, has been demonstrated to preferentially bind super-enhancers [77]. As many oncogenes are found driven by super-enhancers it has been proposed that BET inhibitors could be an epigenetic drug to selectively inhibit inappropriate oncogene expression. Demonstrating how important enhancer activity is to supporting tumor cells, JQ1 and other BET inhibitors have dramatic impacts on tumor cell viability and cell cycle progression in multiple tumor types [82–84]. Shu et al. identified that BET inhibition also holds promise for treatment of triple negative breast cancers for which current treatment options are limited [85].

Supporting the emerging concept of enhancer hijacking in early stage breast cancer, Shen et al. recently reported the tumor suppressor activity of RACK7, a histone reader and component of a demethylase complex with KDM5C. RACK7 ChIP-seq found a strong overlap of RACK7 binding to regions of high H3K4me1 and H3K27Ac occupancy. Analysis with the ROSE (ranking ordering of super enhancers [77, 86]) algorithm, a published algorithm in which enhancers can be ranked by occupancy of common identifiers (Mediator, H3K27ac, H3K4me1, DNase hypersensitivity) found that RACK7 occupied nearly all active super-enhancers in MCF7 and mouse embryonic stem cells. Functionally, RACK7 was found to recruit KDM5C to super-enhancers. Loss of RACK7, or KDM5C, results in loss of H3K4me1, gain of H3K4me3, and increased transcription of associated enhancer targeted genes, including the S100A oncogenes [87]. RACK7 knockout drives tumorigenic phenotypes including increased soft agar growth, invasion, migration, and tumor volume in mammary fat pad xenograft models, suggesting RACK7 functions as a tumor suppressor. Finally, RACK7 expression was examined in 6 patient matched DCIS and IDC samples and was significantly decreased in IDC relative to DCIS [87]. While these observations need to be further validated on a larger patient cohort, together these data suggest that epigenetic chromatin remodeling and enhancer activation as a critical step in the transition to invasive disease.

## Noncoding RNA

Sequencing of the human genome and genome mapping have demonstrated that although greater than 75% of the genome is transcribed, only 2% accounts for protein-coding genes. The remaining RNA falls into several categories including ribosomal, transfer RNA, small nucleolar RNA, microRNA and now recognized long noncoding RNAs. MicroRNAs (miRNAs) are initially transcribed as long primary transcripts (pri-miRNA) by RNA polymerase II. Nuclear Drosha processes pri-miRNA into short hairpins that are then exported to the cytoplasm and processed by Dicer1 into microRNAs of about 19–23 nucleotides long. miRNAs then associate with the RISC complex and target RNA transcripts either with 100% complementarity, leading to transcript degradation, or with imperfect homology to a transcripts 3′ untranslated region effectively inhibiting transcript translation [88, 89]. Thus, one miRNA may target several transcripts and one transcript may be targeted by several miRNAs. Among their targets, miRNAs have been found to regulate, and be regulated by, epigenetic modifiers including HDACs, DNA methyl transferases and Polycomb group proteins [90–92]. Further, given this mechanism of action wherein they regulate gene transcription without impacting the DNA sequence, they are frequently included as epigenetic molecules.

Since the original reporting of miRNA in *Caenorhabditis elegans* in 1993, finding that a short noncoding lin-4 negatively regulated lin-14 [93–95], the field of miRNA has exploded. To date, more than 2500 mature miRNAs have been reported [96]. Dysregulation of miRNAs have been found to influence several aspects of tumorigenesis including differentiation, tumor initiation, and metastasis [97–100]. Indeed, Dicer1 itself may function as a tumor suppressor as inhibition promotes tumorigenesis [101, 102]. Accordingly, global downregulation of miRNAs has been found in many tumor types [103]. Despite the global trend, specific miRNAs have been found to function as tumor suppressors or oncogenes which can be enriched in tumor over normal. Similar to gene analysis by microarray, miRNA expression can also be used to subtype breast cancer [104]. The first examination of miRNAs in pre-invasive lesions was in 2011 where dysregulation of several miRNAs was found relative to normal tissue demonstrating miRNA regulation as a likely contributor to breast cancer progression [105]. Since then expression of a number of miRNAs have been found to play critical roles in breast cancer progression as recently well reviewed [106].

Despite the important role miRNAs are recognized to play, consistent with other epigenetic players, most of the changes are found to occur between normal and DCIS [106, 107]. Increased expression of miR-21 and decreased expression of miR-98 and let-7 is found to distinguish normal tissue and either DCIS or invasive disease [107]. Further, Volinia et al. profiled miRNA expression from 80 IDC, 8 DCIS and 6 normal mammary epithelial samples and found 66 miRNAs differentially expressed between normal and DCIS and only 9 altered in the DCIS to IDC transition (increased let-7d, miR-181a, miR-210, miR-221 decreased miR-10b, miR-126, miR-143, miR-218, miR-335-5p) [108]. Intriguingly, when Hannafon et al. examined miRNAs in reduction mammoplasty samples relative to matched normal/DCIS, 11 miRNAs were differentially expressed between mammoplasty true normal samples and the adjacent normal suggesting a field effect [105]. Together these studies suggest very early changes in miRNA expression drive breast cancer progression, but again these studies are limited by low sample size with limited DCIS representation.

Long noncoding RNAs (lncRNAs) were long discarded as genomic junk as they hold no coding potential. Similar to mRNAs, they are usually transcribed by RNA polymerase II, are broadly classified as any noncoding RNA longer than 200 nucleotides, are generally expressed at very low levels, and are frequently capped, spliced, and polyadenylated. To date over 20,000 lncRNAs have been reported with only a fraction having defined mechanisms of action. Although many lncRNAs are emerging as critical to normal development and cell processes, others may hold no function but be the result of pervasive transcription. Thus, clearly defining if a lncRNA is functional remains one of primary concern in the field [109]. However, lncRNAs have now been identified to function at every level of gene regulation [110]. Intriguingly the largest class of lncRNA functions in epigenetic modifications through associating with chromatin remodeling complexes including PRC1, PRC2 and the LSD1 DNA methylation protein complex. LncRNA epigenetic functions have been well reviewed [111–113]. The importance of lncRNAs to early stage breast cancer progression is emerging.

The lncRNA HOTAIR is found overexpressed in a number of cancers, including breast cancers, and redirects PRC2 and LSD1, impacting global chromatin architecture. HOTAIR expression is known to be elevated in primary breast tumors and depletion can inhibit tumor invasiveness [38, 114]. In depth reviews of HOTAIR function expand on its role in tumorigenesis and metastasis [113, 115], and now HOTAIR expression may be an early indicator of those early lesions that may progress. As previously described, Johnson et al. performed a longitudinal study wherein they compared patient matched normal and DCIS and followup DCIS that progressed to invasive disease [38]. HOTAIR demonstrated increased DNA methylation throughout the gene body associated with progression. HOTAIR expression was also identified by Abba et al. to be upregulated in aggressive, high-grade DCIS cases which were classified as highly proliferative, basal like or ERBB2+ relative to less aggressive, low-grade DCIS cases [12].

Iacoangeli et al. identified the lncRNA BC200 to have increased expression between normal, high-grade DCIS, and highest levels in invasive carcinomas. BC200 expression is normally restricted to neuronal cells where it has been found to function in translation regulation. Importantly, expression of BC200 was found to be greatly increased in high-grade versus low-grade DCIS, as well as invasive malignant breast tissue suggesting it may be useful as a prognostic indicator [116–118]. Eades et al. [119] identified long intergenic noncoding RNA regulator of reprogramming (linc-ROR) expression to increase between normal, DCIS and IDC patient samples as well as between MCF10A normalized breast epithelial cell and breast cancer cell lines (MCF7, MDA-MB-231, Hs578T). Increased expression of lincRNA-ROR sponges miR-145 which increases ARF6 expression, a known regulator of tumor cell invasiveness. Liu et al. identified NF-KappaB Interacting LncRNA (NKILA) to be highly expressed in normal and DCIS samples, but decreased in invasive disease. NKILA interacts with an NFKB:iKB protein complex inhibiting NF-kB nuclear translocation and signaling. Attenuation of NKILA in invasive disease enhances Nf-kB signaling and correlates with poor patient prognosis [120]. Gooding et al. [121] identified several lncRNAs through an in silico approach to be enriched in DCIS relative to adjacent normal (LINC01562, HCG20, SGO1-AS1, PRNCR1, LINC01206, and BORG*).* Expression of the lncRNA BORG (BMP/OP-Responsive Gene) was confirmed in patient samples to be enriched in DCIS over adjacent normal and further, over expression supports proliferation in vitro and in xenograft models collectively.

Currently only a handful of long noncoding RNAs have been found to play a role directly in DCIS, however many are emerging as dysregulated in invasive disease as reviewed elsewhere [113]. UCA1 is a lncRNA found to promote epithelial-mesenchymal transition (EMT) and invasion in breast cancer cells, is found enriched in breast tumors, and correlates with advanced clinical stage together suggesting its expression supports breast tumorigenesis and progression [122, 123]. As their mechanisms continue to be defined, either in epigenetic modification or through features critical to DCIS (proliferation, migration or invasion) more are sure to be discovered. lncRNA discovery in early stage breast lesions is limited in part by the fact that lncRNA expression is generally low relative to mRNAs and therefore may be missed in some RNA-seq protocols. Further, lncRNA annotations are continuing to be expanded and redefined. Future work focused on reannotation of currently available RNA-seq would support current studies that remain limited by sample size, as well as serve as a platform for discovery.

Finally, a new emerging class of noncoding RNA are RNAs that are associated with active enhancers (eRNAs). Active enhancers themselves are found to be transcribed producing either small, nonspliced and short-lived RNAs, or noncoding RNAs more similar to mRNAs that are spliced, capped and polyadentlyated (lnc-eRNAs) [124–126]. Expression of enhancer associated RNAs is highly related to enhancer activity and may serve as a readout to identify active enhancers [127]. Further, eRNAs themselves have been found to contribute to enhancer function. eRNAs have been found to be critical to maintaining open chromatin structure to enhance target gene transcription, initiate and stabilize enhancer-promoter loops, and regulate enhancer activity through both recruiting and evicting transcriptional machinery [128–132]. One such enhancer associated RNA, PRNCR1 was also identified in silico by Gooding et al. to be upregulated in DCIS and triple negative breast cancer relative to normal. As the roles of enhancers in driving breast cancer progression emerges, expression of eRNAs have the potential not only to serve as biomarkers but also as drug targets by which to pinpoint specific dysregulated enhancers.

## Concluding Remarks

**Fig. 1:**
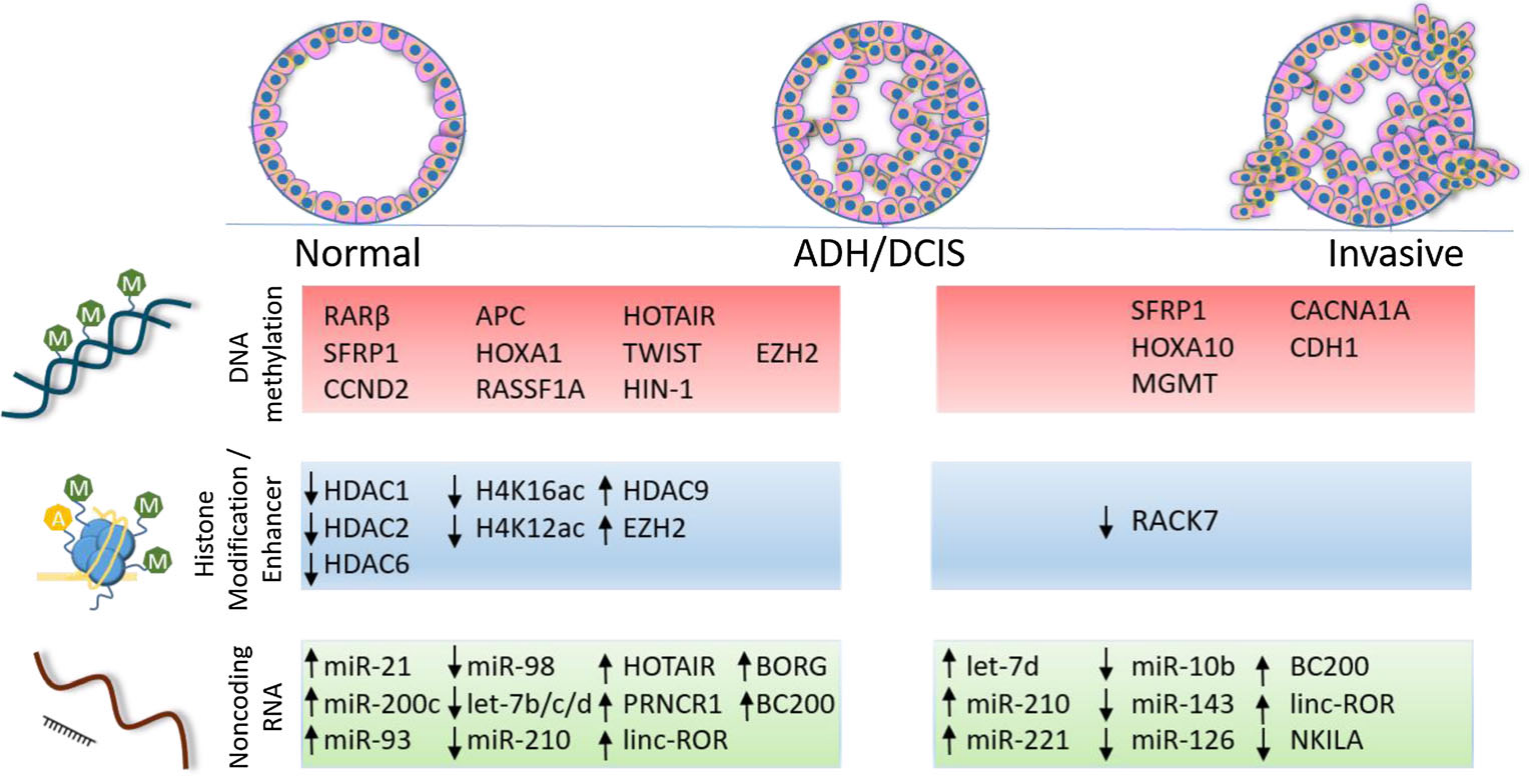
Genes impacted by indicated epigenetic modification are listed where they are found to be dysregulated, either between normal and either atypical ductal hyperplasia (ADH) or ductal carcinoma in situ (DCIS) or between DCIS and invasive ductal carcinoma. All genes listed under DNA methylation demonstrated an increase in methylation in indicated transition

Currently, we are far from a clinically useful understanding of what distinguishes indolent and aggressive DCIS. As with proteomic and transcriptomic studies, the majority of epigenetic changes are found as early events, existing even in the earliest pre-cursor lesions, with far fewer alterations between DCIS and IDC. Despite the similarities between DCIS and IDC lesions, several factors have been identified to be differentially regulated in the transition to invasiveness (Fig. 1, Tables 1 and 2). Although many of these studies are limited by small sample size and have various methodologies for collection (bulk or microdissection), they demonstrate that molecular changes can be defined. Further, a number of genes between DCIS and IDC are found in multiple independent studies, including changes to tumor suppressors (RASSF1A, RARβ), cell cycle related (CCND2) and development genes (Homeobox genes). Most intriguing, polycomb repressive complex target genes as a group have been indicated as early targets in both human patient samples and mouse models of progression. Taken together, evidence suggests that changes to the global chromatin organization, through polycomb targeting and enhancer signaling, driving multiple interconnected pathways, is what drives overall plasticity and progression that may not be captured by looking at individual dysregulated genes.

**Table 1:** Epigenetic modifiers

Epigenetic modifier	Full name	Modification	Function in progression
PRC2	Polycomb repressive complex 2	Histone methylation (H3K27me2 & H3K27me3)	In the transition from DCIS to IDC, there is an enrichment in altered methylation of PRC2 target genes
EZH2	Enhancer of zeste homolog 2	Histone lysine N-methyltransferse catalytic subunit of PRC2	Expression increases between normal, DCIS and IDC
LSD1	Lysine-specific demethylase 1	H3K4 and H3K9 histone methylation eraser	LSD1 expression increases with DCIS grade
HOTAIR	HOX Transcript Antisense RNA	Binds and redirects PRC2 and LSD1 to silence target genes	Expression increases between normal, DCIS and IDC. Likely redirects PRC2
KDM5C	Lysine (K)-Specific Demethylase 5C	Demethylates Lys-4 of histone H3	Functions to regulate enhancer activity
RACK7	Histone reader	Recruits KDM5C to enhancer regions	Expression decreases between DCIS and IDC

Indicated epigenetic modifiers have been implicated in early breast cancer progression. Each has the potential to modify chromatin at hundreds of locations resulting in global chromatin remodeling

**Table 2:** Genes altered during progression

Gene name	Normal to ADH/DCIS	DCIS to IDC	Reversible?	Source
MGMT		Increase DNA methylation	yes	Verschuur-Maes [27]
CACNA1A		Increase DNA methylation	yes	Verschuur-Maes [27]
APC	Increase DNA methylation		yes	Park [28], Hoque [32]
DLEC1	Increase DNA methylation		yes	Park [28]
HOXA1	Increase DNA methylation		yes	Park [28]
SFRP1		Increase DNA methylation	yes	Park [28]
HOXA10		Increase DNA methylation	yes	Park [28]
RASSF1A	Increase DNA methylation		yes	Park [28], van Hoesel [29], Lehhman [30], Fackler [31]
RARβ	Increase DNA methylation		yes	van Hoesel [29], Fackler [31]
CCND2	Increase DNA methylation		yes	Lehhman [30], Fackler [31]
TWIST	Increase DNA methylation		yes	Fackler [31]
HIN-1	Increase DNA methylation		yes	Fackler [31]
CDH1		Increase DNA methylation	yes	Hoque [32]
Homeobox family genes	Differentially methylated	Differentially methylated	yes	Tommasi [37], Johnson [38]
Polycomb repressive complex target genes	Differentially methylated	Differentially methylated	yes	Tommasi [37], Johnson [38], Cai [39]
HOTAIR	Increase DNA methylation, Increase expression		yes	Johnson [38]
CPA1	Increase DNA methylation	Increase DNA methylation	yes	Fleischer [34]
CUL7	Increase DNA methylation	Increase DNA methylation	yes	Fleischer [34]
LRRTM2	Increase DNA methylation	Increase DNA methylation	yes	Fleischer [34]
POU2AF1	Increase DNA methylation	Increase DNA methylation	yes	Fleischer [34]
EZH2	Increased Expression	Increased Expression		Cai [39], Kleer [47], Ding [48]
HDAC1,2,6	Decreased expression			Suzuki [63]
H4K16ac	Decreased		yes	Suzuki [63]
H4K12ac	Decreased		yes	Suzuki [63]
HDAC9	Increased Expression			Lapierre [69]
RACK7		Decreased expression		Shen [87]
PRNCR1		Increased Expression		Gooding [121]
linc-ROR	Increased Expression	Increased Expression		Eades [119]
BORG	Increased Expression			Gooding [121]
NKILA		Decreased expression		Liu [120]
BC200	Increased Expression	Increased Expression		Iacoangeli [116]
miR-21	Increased Expression			Farazi [107], Volinia [108], Hannafon
miR-98	Decreased expression			Farazi [107], Volinia [108], Hannafon [105]
let-7	Decreased expression	Increased Expression		Farazi [107], Volinia [108]
miR-181a		Increased Expression		Volinia [108]
miR-210		Increased Expression		Volinia [108]
miR-221		Increased Expression		Volinia [108]
miR-10b	Decreased expression	Decreased expression		Hannafon [105], Volinia [108]
miR-143	Decreased expression	Decreased expression		Farazi [107], Volinia [108]
miR-126		Decreased expression		Volinia [108]
miR-218		Decreased expression		Volinia [108]
miR-335-5p		Decreased expression		Volinia [108]
miR-200c	Increased Expression			Hannafon [105], Volinia [108]

Many of the genes identified in this review were only identified by one study, underscoring the challenges to understanding the DCIS to IDC transition

Further, many groups have identified epigenetic field-effects in the adjacent normal, demonstrating that the local microenvironment is likely critical to determining invasive potential and limiting the ability to create a decisive progression signature from DCIS and IDC samples alone. Despite these challenges, advanced sequencing technologies will now allow interrogation of the methylome, to better capture and validate changes between DCIS and IDC versus interrogating a small number of candidate genes. Similarly, histone modification studies have been focused on global reader, writer and eraser expression changes. It seems critical to understand where these modifications are taking place. We believe future studies looking at changes to enhancer signaling and global chromatin organization will reveal networks previously unappreciated. To that end, epigenetic targeted therapeutics, such as BET inhibitors may find a place in early treatment of more aggressive DCIS.

With the increased technology supporting critical discovery, the foremost limitations moving forward will be in collecting primary patient samples and the capacity to do longitudinal studies. Given the increased emphasis on understanding DCIS progression, and the advances in sequencing technology, building a signature incorporating epigenetic, genetic and pathological features to determine if a patient requires immediate intervention or simply monitoring, seems within reach.

## Abbreviations

DCIS: Ductal carcinoma in situ
IDC: Invasive ductal carcinoma
ADH: Atypical ductal hyperplasia
HDAC: Histone deacetylase
PRC: Polycomb repressive complexes
CCL: Columnar cell lesions
FEA: Flat epithelial atypia
EZH2: Enhancer of zeste homolog 2
LSD1: Lysine-specific demethylase 1
5-me-C or m5C: 5-methyl-cytosine
HMEC: Human mammary epithelial cells
BET: Bromodomain and extraterminal
